# Prognostic Value of Obesity on Both Overall Mortality and Cardiovascular Disease in the General Population

**DOI:** 10.1371/journal.pone.0127369

**Published:** 2015-05-20

**Authors:** Isabel Ponce-Garcia, Marta Simarro-Rueda, Julio Antonio Carbayo-Herencia, Juan Antonio Divisón-Garrote, Luis Miguel Artigao-Ródenas, Francisco Botella-Romero, Antonio Palazón-Bru, Damian Robert James Martínez-St. John, Vicente Francisco Gil-Guillén, GEVA (Group of Vascular Diseases from Albacete)

**Affiliations:** 1 Casas Ibáñez Primary Health Care Centre, Health Service of Castilla la Mancha, Albacete, Spain; 2 Zona IV Primary Health Care Centre, Health Service of Castilla la Mancha, Albacete, Spain; 3 Department of Clinical Medicine, Miguel Hernández University, San Juan de Alicante, Spain; 4 Cátedra de Riesgo Cardiovascular, San Antonio Catholic University, Murcia, Spain; 5 Zona III Primary Health Care Centre, Health Service of Castilla la Mancha, Albacete, Spain; 6 Endocrinology and Nutrition Service, General Hospital, Albacete, Spain; 7 Castilla La Mancha School of Medicine, Albacete, Spain; 8 Research Unit, Elda General Hospital, Elda, Spain; University of Bologna, ITALY

## Abstract

**Background:**

Obesity represents an important health problem and its association with cardiovascular risk factors is well-known. The aim of this work was to assess the correlation between obesity and mortality (both, all-cause mortality and the combined variable of all-cause mortality plus the appearance of a non-fatal first cardiovascular event) in a general population sample from the south-east of Spain.

**Materials and Methods:**

This prospective cohort study used stratified and randomized two-stage sampling. Obesity [body mass index (BMI) ≥30 kg/m^2^] as a predictive variable of mortality and cardiovascular events was assessed after controlling for age, sex, cardiovascular disease history, high blood pressure, diabetes mellitus, hypercholesterolemia, high-density lipoprotein/triglycerides ratio, total cholesterol and smoking with the Cox regression model.

**Results:**

The mean follow-up time of the 1,248 participants was 10.6 years. The incidence of all-cause mortality during this period was 97 deaths for every 10,000 person/years (95% CI: 80–113) and the incidence of all-cause mortality+cardiovascular morbidity was 143 cases for every 10,000 person/years (95% CI: 124–163). A BMI ≥35 kg/m^2^ yielded a hazard ratio for all-cause mortality of 1.94 (95% CI: 1.11–3.42) in comparison to non-obese subjects (BMI <30 kg/m^2^). For the combination of cardiovascular morbidity plus all-cause mortality, a BMI ≥35 kg/m^2^ had a hazard ratio of 1.84 (95% CI: 1.15–2.93) compared to non-obese subjects.

**Conclusions:**

A BMI ≥35 kg/m^2^ is an important predictor of both overall mortality and of the combination of cardiovascular morbidity plus all-cause mortality.

## Introduction

Obesity is a health problem of great magnitude, impacting importantly on both public health and the economy of affected countries. The 2010 International Obesity Taskforce analysis estimated that 475 million adults worldwide are obese, with this number rising to 1 billion if overweight adults are taken into account as well [1]. The World Health Organization considers obesity to be the “21st century epidemic” [2], and it could even be one of the causes of human life expectancy diminishing for the first time after many decades [3].

A relation between obesity and other cardiovascular risk factors, such as diabetes mellitus, hypertension and dyslipidemia, is well known [4–8]. Moreover, classic studies have shown that obesity is not only related to mortality [9–11], it has also been identified as an independent risk factor for the development of ischemic heart disease, estimating that achieving optimal weight may contribute to a 25% reduction in coronary disease and a reduction of up to 35% in cerebrovascular disease and heart failure [12]. The INTERHEART study found an independent relation between obesity, measured by the waist-to-hip ratio, and the risk of acute myocardial infarction [13]. The EPIC cohort study also revealed that waist circumference and the waist-to-hip ratio are independently associated with all-cause mortality [14]. Thus, the most recent guidelines of the European Society of Cardiology and other societies involved in cardiovascular disease prevention recommend weight loss as one of the goals that could contribute to reducing the incidence of cardiovascular disease [15].

Accordingly, considering that cardiovascular diseases (CVD) represent the main cause of mortality in Spain, as well as in most western countries [15,16], and that 20–30% of cardiovascular mortality can be attributed to overweight [17], there is no doubt that addressing and treating obesity constitutes a main priority. Although some studies have noted a J-shaped curve between obesity and mortality, with greater mortality in high and low categories obtained by measuring the body mass index (BMI) (obesity paradox), the comorbidity associated with excess adiposity could be indicating a “continuum” when present. Nevertheless, it is still unclear what the lowest grade of obesity is that can be considered an independent predictive factor of morbidity and mortality [18]. Studies like the meta-analysis of Flegal et al. and the Prospective Studies Collaboration have found a relation between excess weight and overall mortality. However, these studies did not include data from our geographical area [19, 20]. For this reason, the main purpose of this study was to determine the degree of obesity above which there is an association with mortality and CVD in a general population cohort from the province of Albacete, in south-eastern Spain.

## Material and Methods

### Study population

Persons aged 18 or older from the general population in Albacete (Spain).

### Study design and participants

This prospective cohort study was done in two stages, the first between 1992–1994 and the second between 2004–2006. The sample was drawn from the general population ≥18 years of age in the province of Albacete in the autonomous community of Castilla-La Mancha (south-east Spain). For the study sample to be representative, subjects were randomly recruited from the population census of 1991, which included 22 towns in the province, and stratified and randomized in a two-stage sampling, both stages being proportional to the size of the population included [18]. The second stage, undertaken 10–14 years later, recorded data in the study sample about all-cause mortality, CVD, and persons lost to follow-up. Each participant was followed from recruitment until the development of CVD, death or last contact.

Individuals from the initial population with a body mass index (BMI) <18.5 kg/m^2^ were excluded from the study, because a BMI <18.5 kg/m^2^ can be associated with low-weight disorders and is not therefore considered a normal weight [20].

### Variables and measurements

Outcomes: time-to-death, and time-to-death/CVD (the first occurrence). CVD registered during the follow-up were: any kind of clinically documented angina; myocardial infarction with a clinical report including a concordant enzyme activity pattern, ultrasound and/or angiographic study or basal electrocardiogram allowing its unequivocal localization; stroke in the presence of either permanent and measurable neurological deficits or of neurological symptoms and/or signs which, having been attributed by a physician to a transient ischemic attack and documented in a clinical report, later completely disappeared; peripheral artery disease if patients presented symptoms between Fontaine's grade II and IV (grade I was excluded due to lack of precision). Death was registered from both the clinical records and the official registry of deaths.

Exposure variables: age (years), gender, blood pressure (systolic and diastolic) (mmHg), BMI (kg/m^2^), family and personal history of ischemic heart disease and CVD, glucose (mmol/L), total cholesterol (mmol/L), triglycerides (mmol/L), high-density lipoprotein (HDL) cholesterol (mmol/L), HDL cholesterol/triglycerides ratio, hypertension, diabetes, hypercholesterolemia, hypertriglyceridemia and smoking status.

The definition and collection of these variables has already been described [21]. In summary, a patient was considered to be hypertensive when measures of blood pressure were ≥140/90 mmHg [22], or when the patient was receiving antihypertensive treatment. A patient was deemed to have diabetes when the fasting blood glucose level was ≥7.0 mmol/L (measured twice) [23], or when the subject was under treatment with oral hypoglycemic agents or insulin. Patients were considered to have hypercholesterolemia when the total cholesterol value was >5.17 mmol/L or if they were taking lipid-lowering treatment. The BMI was calculated from measurements of weight and height done under standardized conditions.

The participants were classified into three categories according to their BMI values (kg/m^2^): non-obese group (BMI 18.5–29.9); obese group (BMI ≥30)→ 1) those with Class I obesity (BMI 30–34.9), and 2) those with a BMI ≥35 (Class II [BMI 35.0–39.9] and Class III [BMI ≥40] obesity).

### Sample size

The final cohort sample comprised 1,062 patients. Assuming 95% confidence, an expected censored proportion of 75%, an exposure proportion of 10% and a an expected hazard ratio (HR) of 1.85, the power to contrast a HR different to 1 was calculated. The resulting value was 85.25%.

### Statistical methods

Categorical variables are described in exact quantities and also using percentages; quantitative variables are summarized with both the mean and the standard deviation.

The participants who were lost to follow-up and those who continued throughout the follow-up study were compared using the appropriate statistical test (qualitative variables →chi-square test and Fisher's test; quantitative variables →t test). The Kaplan-Meier estimator was used to calculate approximately the survival probability of the different BMI groups, comparing them through the log-rank test. The Cox regression model was used to identify prognostic variables for the outcomes calculating the adjusted HR according to gender, age, CVD, hypertension, diabetes, hypercholesterolemia, HDL-cholesterol/triglycerides ratio, smoking status and BMI groups. The remaining variables were not introduced in the models due to collinearity issues. We verified that instantaneous hazards were proportional. The proportionality of the instant risks was calculated using the Kaplan-Meier log-log survival curves and assessing the Schoenfeld’s residuals as a statistical test [24]. In hypothesis testing, the maximum rate of type I error was set lower than 5%. The confidence intervals (CI) were calculated for each relevant parameter. Data were analyzed using SPSS (SPSS for Windows, 15.0, SPSS Inc, Chicago, IL).

### Ethical consideration

The study was approved by the ethics committee of the General University Hospital of Albacete and was performed in accordance with the ethical standards laid down in the 1964 Declaration of Helsinki and its later amendments. All the participants gave their written informed consent prior to their inclusion in the study.

## Results

**Fig 1:**
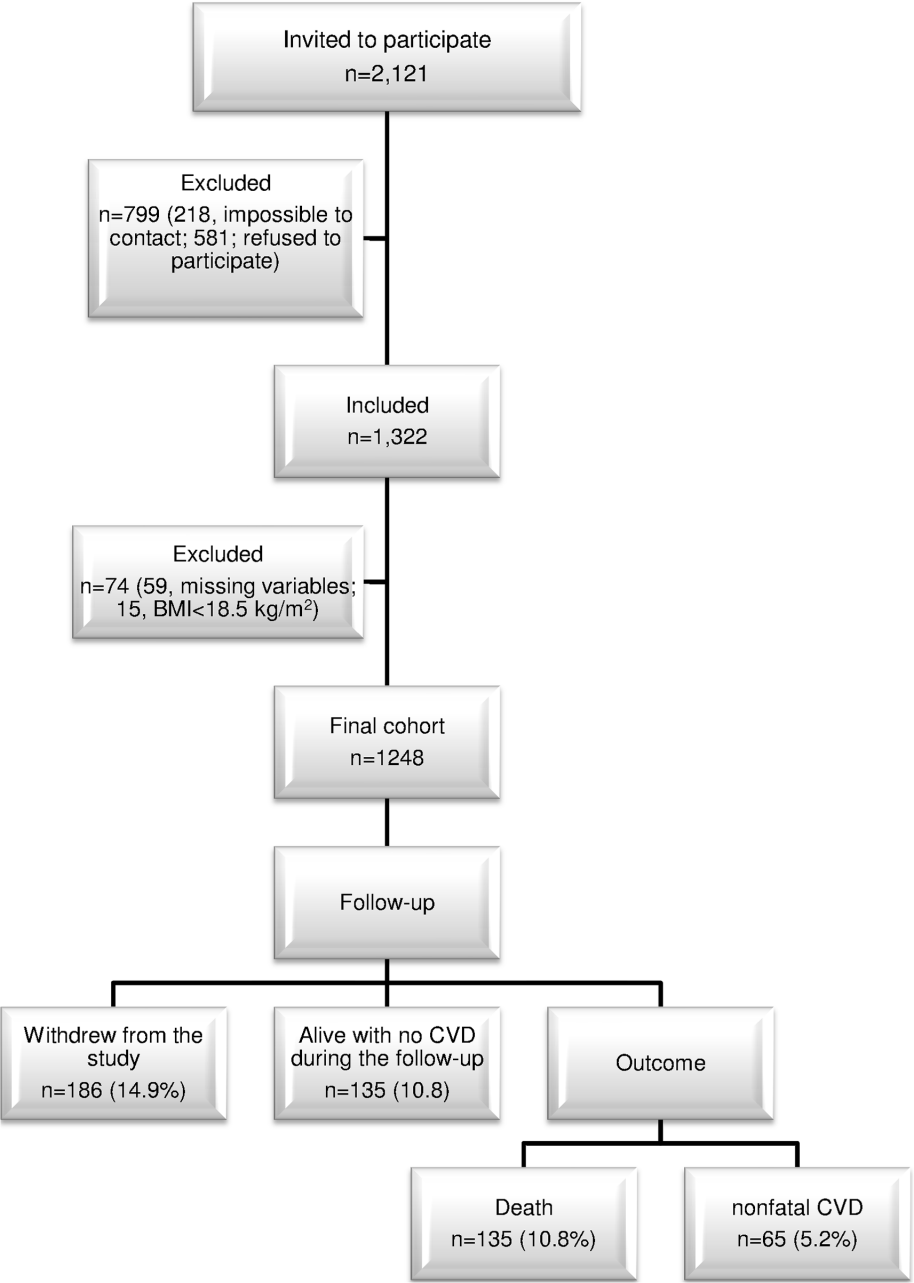
Study sample diagram and main results after an average follow-up of 10.6 years. BMI: body mass index; CI, confidence interval; CVD: cardiovascular disease.

The mean follow-up time of our cohort was 10.6 years. Fig 1 shows the number of patients in each study stage. Of the 2,121 patients invited to participate in the study, 1,322 attended the appointment (response rate: 62.3%). Of those subjects who attended the appointment, 59 were excluded from the study as no venous blood sample could be collected and another 15 whose BMI was <18.5 kg/m^2^. The number of participants in the first stage was therefore 1,248, of whom 186 were lost to follow-up (14.9%, 95% CI: 12.9–16.9%). Participants were considered to be lost to follow-up if they failed to attend for examination on the day of the appointment, failed to reply to two letters sent successively by post and then could not be contacted by telephone.

Due to the low number of participants with a BMI between 35–39.9 kg/m^2^ (65 patients) and a BMI ≥40 kg/m^2^ (23 patients), these were grouped together.

Table 1 shows the characteristics, both of the participants and of the subjects lost to follow-up. No significant differences were observed between them (except for diastolic blood pressure, where no clinically relevant differences were found).

**Table 1:** Characteristics of the participants who were and were not lost to follow-up.

	Not lost to follow-up	Lost to follow-up	
Variables	n = 1,062	n = 186	p-value
Age (years)	49.11 (17.7)	47.18 (18.7)	0.175
Male gender	479 (45.1)	91 (48.9)	0.334
SBP (mmHg)	133.83 (21.7)	130.63 (24.1)	0.068
DBP (mmHg)	81.90 (12.2)	79.93 (13.2)	0.045
BMI (kg/m^2^)	27.71 (4.8)	27.11 (4.4)	0.114
Family history of IC	109 (10.3)	19 (10.2)	0.984
IHD	37 (3.5)	8 (4.3)	0.581
CVD	61 (5.7)	16 (8.6)	0.135
Glucose (mmol/L)	5.6 (1.7)	5.5 (1.3)	0.424
Total cholesterol (mmol/L)	5.2 (1.0)	5.1 (1.1)	0.249
Triglycerides (mmol/L)	1.2 (0.8)	1.1 (0.7)	0.295
HDL cholesterol (mg/dL)	1.2 (0.3)	1.2 (0.3)	0.697
HDL cholesterol/triglycerides ratio	0.62 (0.4)	0.66 (0.4)	0.221
Hypertension	485 (45.7)	74 (39.8)	0.137
Diabetes	113 (10.6)	20 (10.8)	0.963
Hypercholesterolemia	545 (51.3)	91 (48.9)	0.547
Hypertriglyceridemia	175 (16.5)	34 (18.3)	0.544
Low HDL cholesterol	431 (40.6)	77 (41.4)	0.835
BMI groups			0.062
No obesity	762 (71.8)	135 (72.6)	
Class I obesity	218 (20.5)	45 (24.2)	
Class II and III obesity	82 (7.7)	6 (3.2)	
Smoker	328 (30.9)	61 (32.8)	0.604

Quantitative variables are summarized through mean and standard deviation (SD). Categorical variables are expressed as percentages.

SBP: systolic blood pressure; DPB: diastolic blood pressure; BMI: body mass index; IHD: ischemic heart disease; CVD: cardiovascular diseases; HDL: high-density lipoprotein.

During the follow-up period, 200 subjects (16%, 95% CI: 14.0–18.1%) experienced either CVD (65 cases; 5.2%, 95% CI: 4.0–6.4%) or death (135 cases; 10.8%, 95% CI: 9.1–12.5%). Of the deaths, 65 (5.2%, 95% CI: 4.0–6.4%) were due to CVD and 70 (5.6%, 95% CI: 4.3–6.9%) due to other causes, cancer being the most important (28 deaths, 2.2%, 95% CI: 1.4–3.0%), followed by respiratory diseases (12 cases, 0.96%, 95% CI: 0.04–0.15%). The mortality density incidence for all deaths during this period was 97 for every 10,000 person/years (95% CI: 80–113 deaths for every 10,000 person-years) and the combined incidence for the combination of all-cause mortality plus cardiovascular event morbidity was 143 cases for every 10,000 person-years (95% CI: 124–163 outcomes for every 10,000 person-years).

Table 2 reveals that, after controlling for age, gender, hypertension, diabetes, smoking status, HDL-cholesterol/triglycerides ratio, hypercholesterolemia and previous CVD, a BMI ≥35 kg/m^2^ had an instantaneous hazard rate 84% (HR = 1.84; 95% CI: 1.15–2.93, p = 0.011) higher than non-obese patients for the combination of CVD morbidity plus overall mortality. Furthermore, participants with a BMI ≥35 kg/m^2^ had an instantaneous hazard rate 94% (HR = 1.94; 95% CI: 1.11–3.41, p = 0.021) higher than non-obese patients for all-cause mortality.

**Table 2:** Hazard ratios of predictor variables for outcomes.

	Adj. HR		Adj. HR	
	for death/CVD		for death	
Variables	(95% CI)	p-value	(95% CI)	p-value
Male gender	2.36 (1.71–3.27)	<0.001	2.54 (1.70–3.81)	<0.001
Age (5-year increment)	1.60 (1.50–1.70)	<0.001	1.80 (1.65–1.96)	<0.001
Diabetes	1.44 (1.05–1.99)	0.026	1.73 (1.19–2.52)	0.004
Hypertension	1.33 (0.94–1.87)	0.104	1.27 (0.83–1.93)	0.271
Smoker	1.52 (1.05–2.20)	0.027	1.54 (0.98–2.40)	0.061
CVD	1.19 (0.83–1.72)	0.350	1.16 (0.75–1.80)	0.508
Hypercholesterolemia	1.04 (0.77–1.41)	0.780	0.86 (0.60–1.22)	0.388
HDL-c/tryglicerides ratio	0.72 (0.45–1.17)	0.189	0.68 (0.37–1.25)	0.213
BMI				0.037
<30 kg/m^2^	1 (reference)	0.038	1 (reference)	
30–34.9 kg/m^2^	1.09 (0.77–1.54)		0.89 (0.57–1.39)	
≥35 kg/m^2^	1.84 (1.15–2.93)		1.94 (1.11–3.42)	

Adj. HR: adjusted hazard ratio; CI: confidence interval; CVD: cardiovascular disease; HDL-c: high-density lipoprotein cholesterol; BMI: body mass index. Control variables in all models were age, gender, diabetes, hypertension, smoking status, personal history of cardiovascular disease, hypercholesterolemia, high-density lipoprotein cholesterol/triglycerides ratio and BMI.

**Fig 2:**
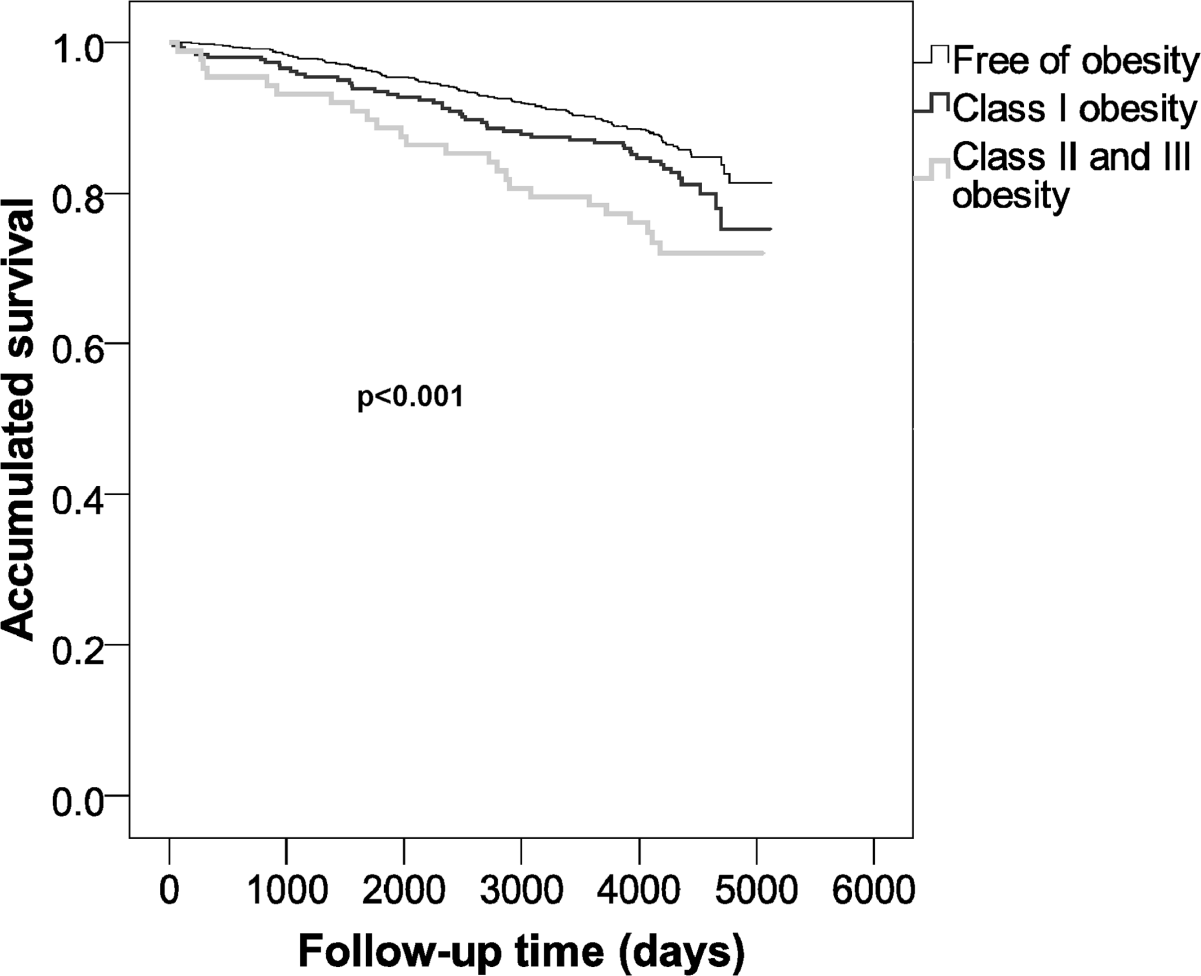
Survival probability for either cardiovascular morbidity or overall mortality between obesity classes.

**Fig 3:**
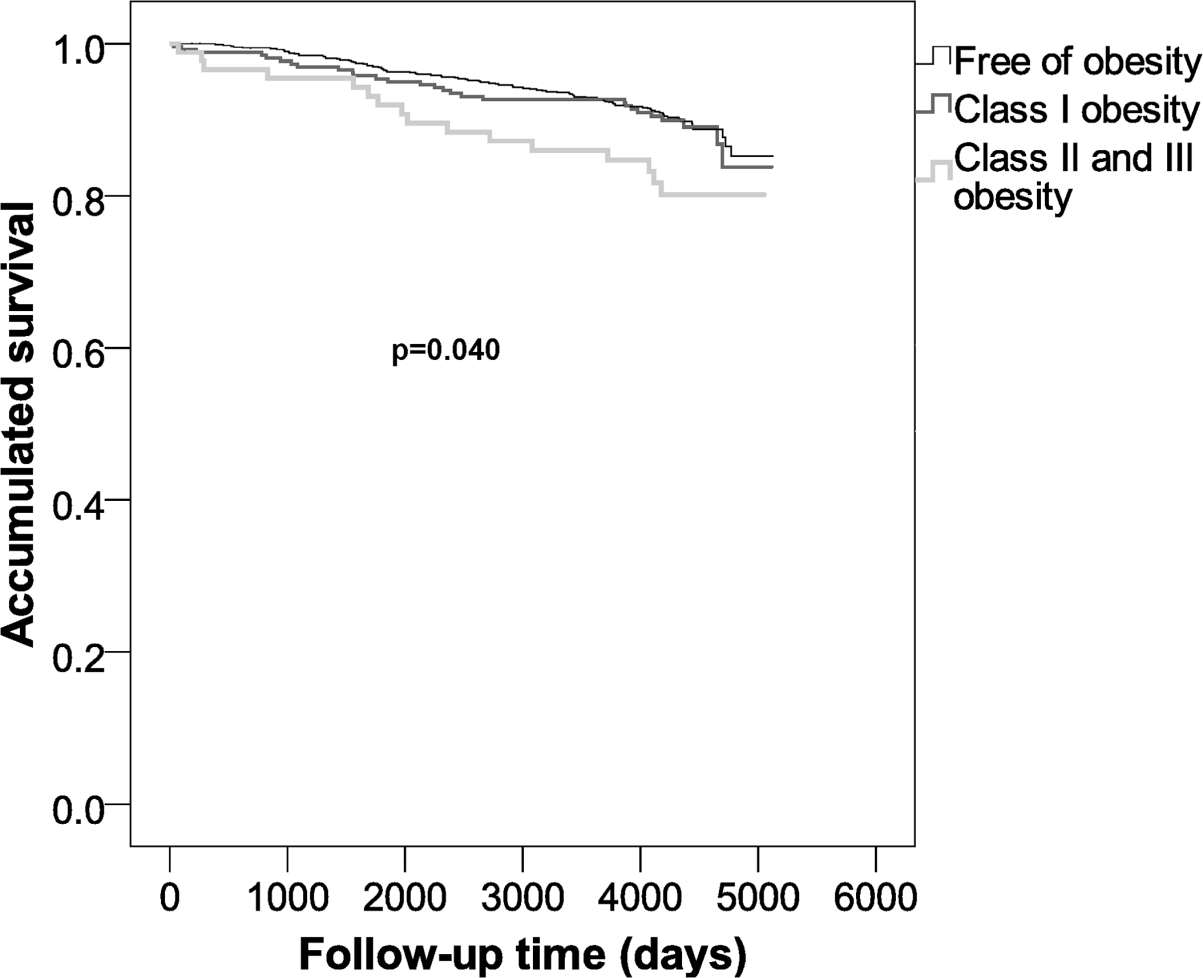
Survival probability when assessing overall mortality for obesity classes.

Finally, Figs 2 and 3 show a significant difference between the BMI groups and time-to-outcomes (death/CVD, p<0.001; death, p = 0.040).

## Discussion

Regarding the aim of our study, there was a statistically relevant relationship between our outcomes and the three groups defined according to the BMI (<30, 30–34.9, and ≥35 kg/m^2^).

The probability of survival was lower for obese participants, declining with the increase in obesity grade, for the combination of all-cause mortality plus non-fatal CVD, and for overall mortality. Controlling for age, gender, previous CVD, hypertension, diabetes, smoking, hypercholesterolemia, HDL-cholesterol/triglycerides ratio, showed that the incidence rate of both the above-mentioned combination and of overall mortality was higher in those participants with higher grades of obesity. In our population, therefore, a BMI ≥35 kg/m^2^ was an important risk factor both for overall mortality and for the combination of all-cause mortality plus CVD.

Recent studies, like the meta-analysis carried out by Flegal et al. [19], yielded very similar results to ours, observing that, in comparison to normal- weight persons, obesity in general and particularly a BMI ≥35 kg/m^2^ were associated with an increase in all-cause mortality (HR 1.18 [95% CI: 1.12–1.25] for obesity in general, and HR 1.29 [95% CI: 1.18–1.41] for a BMI ≥35 kg/m^2^). A BMI between 30–34.9 kg/m^2^ was not associated with an increase in mortality (HR 0.95, 95% CI: 0.88–1.01), suggesting that the excess mortality in obese patients is owing to those with a higher BMI. The study performed by the VHP&PP group in a cohort of 184,697 Australian adults also found that a BMI >35 kg/m^2^ was associated with a higher mortality (HR: 2.13 in males and 1.60 in females), especially from cardiovascular events [25]. The Prospective Studies Collaboration group analyzed mortality in 57 prospective studies carried out in Europe and North America, finding that BMI was a strong predictor of overall mortality, such that with a BMI >25 kg/m^2^, each increase of 5 kg/m^2^ amounted to an increased HR of 1.29 for overall mortality [20]. The study by Faeh et al. found a higher mortality in subjects with a BMI >30 kg/m^2^ (HR: 1.41), mainly from cardiovascular events (HR: 2.05) [26]. The EPIC study established that, besides BMI, other measures of regional adiposity, like the waist circumference or the waist-to-hip ratio, were also related with the increase in overall mortality [27].

### Study limitations and strengths

A notable finding was that the patients who had Class I obesity (BMI 30–34.9) experienced no differences in the outcomes studied as compared with the non-obese population. This could be due to the lack of confounding variables included in the analysis, such as physical activity [28], as these data were not available when the study was originally designed, though other more relevant variables that could explain CVD were included. Further studies, therefore, should also consider additional variables.

As we did not have a large enough sample size, we were unable to assess cardiovascular morbidity and cardiovascular mortality separately. This, too, remains to be done in future studies. Nevertheless, the outcomes analyzed have great clinical relevance and the sample size in this study was sufficient for the analyses desired (statistical power greater than 85%) [29].

Another possible limitation of our study could be the few deaths and cardiovascular events experienced in our sample. However, as mentioned, the sample size was sufficient for the aims, and the sample was obtained randomly, giving great external validity to the results. Finally, to minimize any information bias, calibrated devices were used.

### Conclusion

In our population, obesity is an important predictor of both overall mortality and the combination of cardiovascular morbidity plus all-cause mortality. We, like others [30], therefore believe that prevention and treatment of obesity should be a high priority for the national health system.

Further studies will be necessary to assess and confirm the relation of the different grades of obesity to morbidity and mortality.
